# Viewing heterospecific facial expressions: an eye-tracking study of human and monkey viewers

**DOI:** 10.1007/s00221-019-05574-3

**Published:** 2019-06-05

**Authors:** Kun Guo, Zhihan Li, Yin Yan, Wu Li

**Affiliations:** 1 School of Psychology, University of Lincoln, Lincoln, LN6 7TS UK; 2 State Key Laboratory of Cognitive Neuroscience and Learning, and IDG, Beijing Normal University, Beijing, 100875 China

**Keywords:** Facial expression, Gaze behaviour, Eye tracking, Rhesus macaques, Humans

## Abstract

Common facial expressions of emotion have distinctive patterns of facial muscle movements that are culturally similar among humans, and perceiving these expressions is associated with stereotypical gaze allocation at local facial regions that are characteristic for each expression, such as eyes in angry faces. It is, however, unclear to what extent this ‘universality’ view can be extended to process heterospecific facial expressions, and how ‘social learning’ process contributes to heterospecific expression perception. In this eye-tracking study, we examined face-viewing gaze allocation of human (including dog owners and non-dog owners) and monkey observers while exploring expressive human, chimpanzee, monkey and dog faces (positive, neutral and negative expressions in human and dog faces; neutral and negative expressions in chimpanzee and monkey faces). Human observers showed species- and experience-dependent expression categorization accuracy. Furthermore, both human and monkey observers demonstrated different face-viewing gaze distributions which were also species dependent. Specifically, humans predominately attended at human eyes but animal mouth when judging facial expressions. Monkeys’ gaze distributions in exploring human and monkey faces were qualitatively different from exploring chimpanzee and dog faces. Interestingly, the gaze behaviour of both human and monkey observers were further affected by their prior experience of the viewed species. It seems that facial expression processing is species dependent, and social learning may play a significant role in discriminating even rudimentary types of heterospecific expressions.

## Introduction

Because a significant part of emotional expressions are achieved through movements of facial muscles, facial expressions provide crucial visual cues for humans and a range of non-human mammal species to understand other’s emotional state and intention. The ability to recognize an individual’s expression accurately and quickly plays a crucial role in an animal’s social communication and even survival (Darwin 1872). Consequently, humans are extremely sensitive to each other’s facial expressions. We show inborn predisposition to process expressive facial cues, and the relevant cognitive and perceptual capacities are quickly perfected through increasing practice and exposure over time (Bruce and Young 2012). Although the capability of categorizing conspecific facial expressions, particularly of human viewers, and its underlying cognitive mechanisms are well researched, little is known about the processes in perceiving heterospecific facial expressions. Even though it has been reported that humans can recognize some facial expressions in monkeys (Marechal et al. 2017) and dogs (Tam and Gallagher 2009; Wan et al. 2012), and some basic human facial expressions (e.g. happy vs anger) can be discriminated by monkeys (Kanazawa 1996), dogs (Müller et al. 2015; Albuquerque et al. 2016**)**, horses (Proops et al. 2018), goats (Nawroth et al. 2018) and giant pandas (Li et al. 2017), we still do not know whether the same cognitive process (e.g. face-viewing gaze allocation) is adopted to process conspecific and heterospecific facial expressions in these species.

It has been argued that human perception of basic conspecific facial expressions is a “universality” process (Ekman et al. 1969; Ekman 1994), in which we do not have to learn to recognize basic or universal emotions (e.g. happy, sad, anger, fear, disgust, surprise) from people of different cultures and we interpret other people’s facial expressions according to how we would feel if we looked that way (Ekman and Cordaro 2011). As congenitally blind individuals could produce recognizable facial expressions even though they have never seen other people’s expressions to imitate (Tröster and Brambring 1992) and newborn infants could visually discriminate expressions such as happiness, sadness and surprise (Field et al. 1982), we may have inborn predisposition to the production and perception of (at least) some facial expressions. This “universality” view is further supported by stereotypical and heritable nature of common facial expressions which can be examined through the quantification of facial muscle movements and their composition into different expression categories via the Facial Action Coding System (FACS; Ekman et al. 1975). For instance, a typical happy face is correlated with raised inner eyebrows, cheek and upper lip, and tightened lower eyelid; and an angry expression comprises lowered eyebrows, eyes wide open with tightened lower lid, lips exposing teeth and stretched lip corners (Ekman et al. 1975; Kohler et al. 2004). As each of these basic facial expressions has one or more action units linked to key internal facial features such as eyes, nose and mouth, different local facial features can subsequently transmit expression-specific diagnostic information for ‘universal’ recognition of common expressions in humans (e.g. eyes and mouth contain crucial cues for detecting angry and happy expressions, respectively) (Calvo and Nummenmaa 2008; Smith et al. 2005). Indeed, recent eye-tracking studies have observed that when categorizing facial expressions, we tend to look more often at local face regions that are most characteristic for each expression category (Eisenbarth and Alpers 2011; Guo 2012, 2013; Guo and Shaw 2015), suggesting that gaze allocation at the eyes, nose and mouth regions could be systematically influenced by the viewed facial expressions.

It is plausible that such “universality” process could also be involved in perceiving heterospecific facial expressions, as the homology in emotional behaviours, in facial musculature and in neural system sub-serving emotion expression and perception across mammals (Darwin 1872; Leopold and Rhodes 2010; Schirmer and Adolphs 2017) implies that (at least) some basic facial expressions might be recognizable or understandable across species. Specifically, humans use 17 facial muscle pairs to display expressions and we share this facial musculature fully with great apes and partially with other mammal species (Müri 2016). This commonality in facial anatomy plan could lead to subsequent expression similarities between species. For instance, humans, non-human primates and dogs use zygomaticus major to retract mouth corners and expose teeth. These zygomaticus activities together with jaw dropping represent a joyful display across these species (Parr et al. 2010; Schirmer et al. 2013). Therefore, if different species produce the same or similar facial movements in response to the same emotional event, then individuals might be able to form an understanding of heterospecific emotion through their own facial actions without the need of prior experience of interacting with heterospecifics. Consequently, human viewers may show comparable recognition accuracy and gaze behaviour when judging human, chimpanzee and monkey facial expressions.

On the contrary, “social learning” process argued that facial expressions might be evolutionary by-products of a general-purpose meaning-inference system, and therefore are learned species-specific cultural symbols rather than innate, prototypic and universal communication signals (Barrett 2011). If so, then an associative learning between external events and motivational states is likely needed to understand heterospecific emotions. Recently, this view has motivated a few empirical studies with contradictory findings. For example, in comparison with human expressions, we are less accurate in judging non-human, such as dog, facial expressions. While some studies found recognition accuracy for some expressions (e.g. fearful, playful) were improved with prolonged experience with dogs (Tam and Gallagher 2009; Wan et al. 2012), others argued that experience had very limited role in recognizing dog facial expressions, with experts and non-experts making similar categorization errors and biases (Bloom and Friedman 2013).

Recent comparative eye-tracking studies on humans and non-human primates have shed light on the relative contribution of “universality” and “social learning” processes in attending conspecific and heterospecific social or emotional scenes involving faces. For instance, humans and rhesus monkeys showed comparable tendency to gaze at ‘high-interest’ targets (e.g. expressive human or animal faces) in dynamic video clips (Berg et al. 2009; Shepherd et al. 2010) and static human–human or animal–animal social interaction images (McFarland et al. 2013), and such interspecies gaze correlations were driven by biologically relevant social or emotional cues rather than low-level image properties, such as local image contrast or structure (Shepherd et al. 2010). Furthermore, when presented with a conspecific or heterospecific face alone picture (i.e. without body and background scene) with neutral facial expression, both humans and monkeys tended to show a face-specific directional gaze bias towards the left hemiface (Guo et al. 2009) and stereotypical gaze allocation at socially informative local facial features with a strong preference towards the eyes (Guo et al. 2006; Dahl et al. 2009; Méary et al. 2014). These similarities in social attention behaviour (i.e. strong gaze preference at conspecific or heterospecific faces in social scenes, and at the eye region) have also been observed between humans and chimpanzees (Kano and Tomonaga 2009, 2010) and between other closely related primate species, such as rhesus monkeys, bonobos, orangutans, chimpanzees and gorillas (Kano et al. 2012, 2015, 2018). It seems that humans and some non-human primates are broadly tuned to the same local visual cues when processing social and emotional scenes, suggesting a close evolutionary connection in the organization of their visual system and a ‘universal’ process in social cognition.

In spite of the striking similarities in social attention between humans and non-human primates, these studies have often reported substantial quantitative differences in face-viewing gaze allocation that are species specific and context dependent (e.g. humans tend to gaze at the human eyes more often than the monkey eyes) (Shepherd et al. 2010; McFarland et al. 2013; Kano et al. 2018), implying a possible contribution of “social learning” in face viewing. Furthermore, as these earlier studies are not designed to compare gaze behaviour in viewing of facial expressions of different categories and different species, we do not know the extent to which the “universality” and “social learning” processes are involved in interspecies emotion perception.

To further explore these contrasting views specifically in perceiving conspecific and heterospecific facial expressions, in two separate eye-tracking experiments we examined face-viewing gaze allocation of human (including dog owners and non-owners) and monkey participants while exploring human, chimpanzee, monkey and dog faces with positive (happy/playful/appeasement), relaxed (neutral) and negative (angry/threatening) expressions. It is plausible that both the “universality” and “social leaning” processes could be reduced in perceiving heterospecific facial expressions as they are less biologically relevant to the viewers. To maximally promote and also distinguish between these two processes, we only presented the very basic high-intensity angry and happy conspecific and heterospecific facial expressions, as these expressions are likely to be homologous in morphological features and social functions across the presented species (Leopold and Rhodes 2010; Schirmer and Adolphs 2017), and humans need little learning or experience to recognize high-intensity human happy and angry expressions as suggested by developmental and cross-culture studies (Gao and Maurer 2010; Yan et al. 2016). We also explicitly requested human viewers to categorize the perceived expressions using a free-labelling task, and tested both dog owners and non-owners to further compare the role of prior experience in differentiating dog facial expressions.

However, although the static images of appeasement and silent bared-teeth display in chimpanzee and monkey faces have been proposed as the possible primate homologues of smiling (van Hooff 1972), recent research has indicated that they are ambiguous in social functioning. These facial displays could occur in both aggressive and affiliative situations for the purpose of increasing the likelihood of affiliative behaviour, and hence represent different internal or emotional state in non-human primates (Waller and Dunbar 2005), whereas play face (relaxed open-mouth face) is almost exclusively displayed in positive social context (Parr et al. 2007). Even though both displays may have similar evolutionary function of social bonding and have converged into happy expression in humans (Waller and Dunbar 2005), the ambiguity in appeasement and silent bared-teeth display would lead to difficulty to judge whether humans’ emotion categorization of these face images are correct or not. Taking this into consideration, our final stimuli set included human and dog faces with positive, relaxed and negative expressions; but chimpanzee and monkey faces only with relaxed and negative expressions.

Based on our stimuli and experimental design, the “universality” process would predict that human viewers demonstrate comparable categorization accuracy to the same expression displayed by different species, both dog owners and non-owners show similar expression categorization accuracy for faces of different species, and both human and monkey viewers display similar expression-dependent gaze distribution regardless of the viewed face species (e.g. when viewing the same expression displayed by different species, viewers direct similar amount of fixations at those informative internal facial features, such as eyes, nose and mouth, which transmit diagnostic expressive cues). In contrast, the “social learning” process would predict an experience-dependent expression categorization accuracy in human viewers (e.g. humans show higher categorization accuracy for human faces than for faces of other species, and dog owners show higher categorization accuracy for dog faces than non-owners), and a species-dependent face-viewing gaze distribution in both human and monkey viewers (e.g. different levels of experience may modify amount of attention directed at the eyes in ‘angry’ human, chimpanzee, monkey and dog faces).

## Experiment 1: humans viewing heterospecific facial expressions

### Materials and methods

Fifty-six Caucasian undergraduate participants (20 male, 36 female), age ranging from 18 to 26 years with a mean of 19.88 ± 1.27 (mean ± SD), volunteered to participate in this study conducted at the University of Lincoln. This sample size was determined based on previous research in the same field and was comparable to the published reports (e.g. Kano and Tomonaga 2010; Guo 2012, 2013; Gavin et al. 2017). All participants had normal or corrected-to-normal visual acuity and did not have frequent contact with chimpanzees or monkeys. Twenty-eight participants (19 female, mean age 19.79 ± 1.45) were dog owners with between 1 and 21 years’ experience of dog ownership (mean years of experience 10.39 ± 6.05). The other 28 (17 female, mean age 19.96 ± 1.07) were non-dog owners (i.e. individuals who never had dogs). The Ethical Committee in School of Psychology, University of Lincoln, approved this study. Written informed consent was obtained from each participant prior to the testing, and all procedures complied with the British Psychological Society “Code of Ethics and Conduct”.

Digitized greyscale face images were presented through a ViSaGe graphics system (Cambridge Research Systems, UK) and displayed on a high-frequency non-interlaced gamma-corrected colour monitor (30 cd/m^2^ background luminance, 100 Hz frame rate, Mitsubishi Diamond Pro 2070SB) with the resolution of 1024 × 768 pixels. At a viewing distance of 57 cm, the monitor subtended a visual angle of 40 × 30°.

**Fig. 1:**
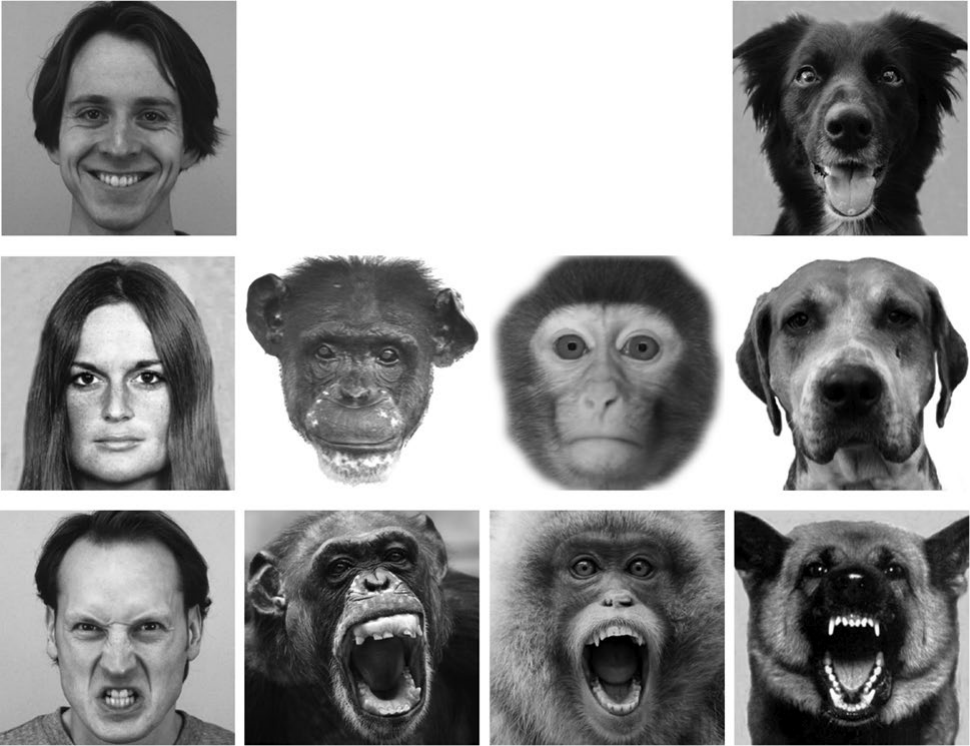
Example of the used stimuli. From left to right: human faces, chimpanzee faces, monkey faces and dog faces displaying positive, relaxed/neutral and negative facial expressions

Unfamiliar face images in full frontal view from four species were used as stimuli (see examples in Fig. 1): 18 human (Caucasian) faces, 12 chimpanzee (*Pan troglodytes*) faces, 12 monkey (9 Rhesus macaques and 3 Japanese macaques) faces and 18 dog (*Canis familiaris*) faces. Human faces were selected from the “Pictures of Facial Affect” (Ekman et al. 1975) and Karolinska Directed Emotional Faces CD ROM (Lundqvist et al. 1998). Chimpanzee, monkey and dog faces were collected from different online sources and own databases (Guo et al. 2009; Racca et al. 2012). The faces of each species were divided into either two or three emotional categories (6 faces per category, see also the justification in “Introduction”) corresponding to the valence of the facial expression displayed: positive (happy expression in human and dog faces), relaxed neutral (close-mouth relaxed faces without obvious facial muscle tension in human, chimpanzee, monkey (5 rhesus macaques and 1 Japanese macaque) and dog faces), and negative (angry expression in human faces; open-mouth threat in chimpanzee and monkey (4 rhesus macaques and 2 Japanese macaques) faces; threatening aggressive facial signals in dog faces). The selection of emotional valence for chimpanzee and monkey faces was guided by criteria defined in ChimpFACS (Parr et al. 2007) and MaqFACS (Parr et al. 2010). The ‘happy’ dog face pictures were taken when presenting dogs with food and talking to them using ‘doggerel’ (similar to ‘baby speech’ but directed to dogs; Mitchell 2001), and the typical ‘happy’ facial reaction was a relaxed face with an open mouth, the tongue out and erect ears (Racca et al. 2012). The ‘threatening’ dog face pictures were taken and chosen from dogs displaying typical aggressive facial signals (e.g. bared teeth, wrinkled muzzle, erect and forward pointing ears), including police dogs trained to display such behaviour (Racca et al. 2012). Furthermore, the emotional valence of non-human faces was further confirmed by two specialists in primatology and in veterinary behavioural medicine. All images shared similar spatial facial configurations, were gamma corrected to ensure a natural shades appearance as seen by human eyes and were displayed once in a random order at the centre of the screen with a resolution of 500 × 500 pixels (18 × 18°).

During the experiment, the participants sat in a chair with their head restrained by a chin rest and viewed the display binocularly. To calibrate eye movement signals, a small red fixation point (FP, 0.3° diameter, 15 cd/m^2^ luminance) was displayed randomly at one of nine positions (3 × 3 matrix) across the monitor. The distance between adjacent FP positions was 10°. The participant was instructed to follow the FP and maintain fixation for 1 s. After the calibration procedure, the participant pressed the response box to initiate a trial. The trial was started with an FP displayed 10° left or right to the screen centre to minimize central fixation bias (Tatler 2007). If the participant maintained fixation for 1 s, the FP disappeared and a face image was presented at the centre of the monitor for 3 s. During the free-viewing presentation, the participant was instructed to “view the face as you normally do” and use one word to label the perceived facial expression. The experimenter then typed the verbal report into the customer-made software. No reinforcement was given during this procedure.

Horizontal and vertical eye positions from the self-reported dominant eye (determined through the Hole-in-Card test or the Dolman method if necessary) were measured using a Video Eyetracker Toolbox (a camera-based system tracking pupil centre and corneal reflection) with 250 Hz sampling frequency and up to 0.25° accuracy (Cambridge Research Systems, UK). The software developed in Matlab computed horizontal and vertical eye displacement signals as a function of time to determine eye velocity and position. Fixations were then extracted from the raw eye-tracking data using velocity (less than 0.2° eye displacement at a velocity of less than 20°/s) and duration (greater than 50 ms) criteria (Guo et al. 2006).

While determining fixation allocation within key internal facial features (i.e. eyes, nose, and mouth), a consistent criterion was adopted to define boundaries between local facial features for different faces (for details and regions of interest examples see Fig. 1 in Guo 2012). Specifically, the ‘eye’ region included the eyes, eyelids and eyebrows; the ‘nose’ or ‘mouth’ region included the main body of the nose or mouth and immediate surrounding area (up to 0.5°). The division line between the mouth and nose regions was the midline between the upper lip and the bottom of the nose. Each fixation was then characterized by its location among feature regions and its time of onset relative to the start of the trial, and the number of fixations directed at each feature was normalized to the total number of fixations sampled in that trial.

The participants’ verbal reports of the perceived facial expressions were analysed off-line. For the positive facial expressions, the correct categorization was accepted for labels such as happy, cheerful, thrilled, playful, pleased, cheeky, laughing and excited. The accepted labels for the relaxed expressions included neutral, dull, blank, bored, clam and relaxed. The accepted labels for the negative expressions included anger, annoyed, upset, mad, threatening, displeased and violent. The label was considered as incorrect if the reported emotional valence was opposite to the presented facial expression valence.

### Results and discussion

To examine the extent to which human expression categorization performance was affected by dog ownership, the viewed face species and facial expressions, we conducted a general linear model (GLM) analysis with expression categorization accuracy as the dependent variable. Only significant main and interaction effect was reported.

**Fig. 2:**
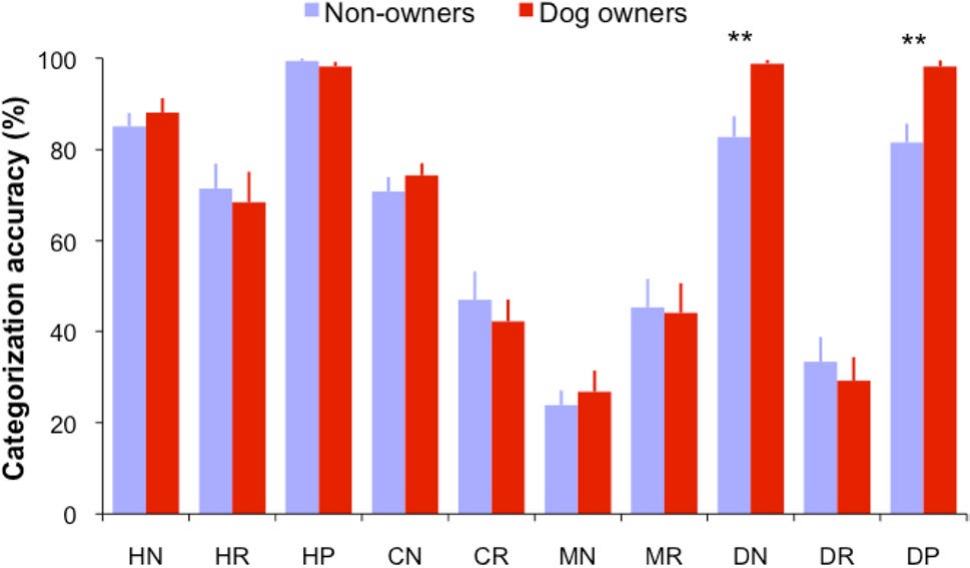
Mean expression categorization accuracy from dog owners and non-owners when labelling human, chimpanzee, monkey and dog facial expressions. *HN* human negative, *HR* human relaxed, *HP* human positive, *CN* chimpanzee negative, *CR* chimpanzee relaxed, *MN* monkey negative, *MR* monkey relaxed, *DN* dog negative, *DR* dog relaxed, *DP* dog positive. Error bars represent SEM. *p < 0.05, **p < 0.01

Regardless of dog ownership, human facial expression categorization performance varied according to the viewed face species and facial expressions [face species: *F*(3,560) = 65.57, *p* < 0.001, *η*_p_^2^ = 0.27; expression type: *F*(2,560) = 88.29, *p* < 0.001, *η*_p_^2^ = 0.25; face species × expression type: *F*(4,560) = 41.36, *p* < 0.001, *η*_p_^2^= 0.24; Fig. 2]. Specifically, for relaxed (neutral) expressions, humans showed the highest categorization performance for human faces (HR: 70% ± 5), the lowest performance for dog faces (DR: 31% ± 4; Bonferroni correction for multiple comparisons, all *p*s < 0.001) and indistinguishable categorization performance for chimpanzee and monkey faces (CR vs MR: 45% ± 5 vs 45% ± 5, *p *=0.99). For negative expressions, they showed the highest categorization performance for dog and human faces (DN vs HN: 91% ± 2 vs 87% ± 2, *p *=0.16), followed by chimpanzee faces (CN: 73% ± 2), and then by monkey faces (MN: 25% ± 3; MN vs CN or HN or DN, all *p*s < 0.001).

Furthermore, when labelling human expressions, human participants were the most accurate in labelling positive expressions (99% ± 1), followed by negative expressions (87% ± 2) and then relaxed expressions (70% ± 5; all *p*s < 0.001). When labelling dog expressions, humans showed the same accuracy in categorizing positive and negative expressions (90% ± 2 vs 91% ± 2, *p *=0.80), which was significantly higher than that for relaxed expressions (31% ± 4; all *p*s < 0.001). Interestingly, humans were more accurate in labelling chimpanzee negative than relaxed expressions (73% ± 2 vs 45% ± 5, *p* < 0.001), but were less accurate in labelling monkey negative than relaxed expressions (25% ± 3 vs 45% ± 5, *p* < 0.001).

Although dog ownership did not show significant impact on overall expression recognition accuracy [*F*(1,560) = 2.19, *p* = 0.14, *η*_p_^2^ = 0.004], dog owners were more accurate in detecting dog negative or positive expressions than non-owners [negative: 99% ± 1 vs 83% ± 5, *t*(54) = − 3.46, *p* = 0.001, 95% CIs (− 25.39, − 6.75), Cohen’s *d* = 0.92; positive: 98% ± 1 vs 82% ± 4, *t*(54) = − 3.86, *p* < 0.001, 95% CIs (− 25.44, − 8.06), Cohen’s *d* = 0.92]. There was no correlation between the length of dog ownership and owners’ categorization performance for dog expressions (two-tailed Pearson correlation for each dog expression, all *p*s> 0.05), probably due to their ceiling level of expression categorization accuracy.

For those expressions with relatively poor categorization accuracy (< 80%), we computed confusion matrices to illustrate which expressions were mistaken for others. For each displayed expression with incorrect labelling, we calculated the percentage of the trials in which participants wrongly categorized the expression using a particular label and reported the commonly confused labels and their percentages. As shown in Table 1, when making mistakes, both dog owners and non-owners tended to label human or dog neutral expression as sad, chimpanzee neutral expression as sad or happy, and chimpanzee or monkey negative expression as happy or surprise. Although less frequently, non-owners were more likely to mislabel dog positive expression as neutral (77%) and negative expression as neutral (26%), happy (22%) or fear (22%).

**Table 1:** Confusion matrices of expression categorization: percentage of participants selecting a given expression label in incorrect trials, averaged across the stimuli set and participants

Displayed expression	Labelled expression	
	Dog owners	Non-owners
Human neutral	Sad (80%)	Sad (79%)
Chimpanzee negative	Happy (54%), surprise (14%)	Happy (58%), surprise (15%)
Chimpanzee neutral	Sad (45%), happy (40%)	Sad (51%), happy (30%)
Monkey negative	Surprise (62%), happy (24%)	Surprise (60%), happy (25%)
Monkey neutral	Sad (60%), happy (12%)	Sad (58%), happy (15%)
Dog neutral	Sad (79%)	Sad (84%)

Considering that the constant face presentation duration across different face images would attract similar amount of fixations or viewing time per trial, and the analysis of fixation and viewing time would lead to qualitatively identical face-viewing gaze distribution in both human (e.g. Guo et al. 2010; Guo 2012) and monkey viewers (e.g. Guo et al. 2003), for eye-movement data analysis we focused on fixation or gaze pattern analysis. Furthermore, previous studies have demonstrated when exploring either relaxed or expressive human faces or non-human animal faces with human-like facial configurations, the vast majority of fixations were allocated at three key internal facial features (i.e. eyes, nose, and mouth) regardless of task demand (e.g. Guo et al. 2010, 2012; Gavin et al. 2017). As these three local facial features can transmit expression-specific diagnostic information for expression recognition (at least) in humans (Smith et al. 2005), our gaze allocation at them are systematically influenced by the viewed facial expressions (Guo 2012, 2013). Taken all together, our analysis of face-viewing gaze behaviour was focused on those fixations directed at the eyes, nose and mouth regions.

**Fig. 3:**
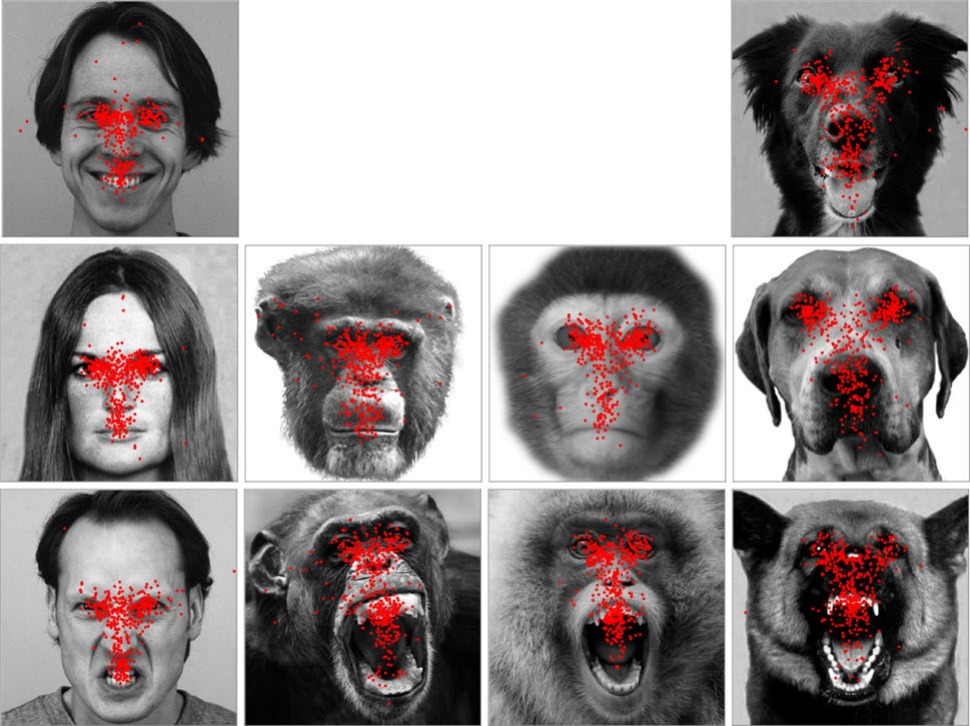
Example of overall face-viewing fixation distribution on these faces. The red dots within each face image indicate the position of each fixation sampled during this face-viewing trial across all human participants

As shown in the face examples in Fig. 3, in agreement with previous studies, our analysis revealed that during the face exploration, human viewers allocated the vast majority of fixations at the eyes, nose and mouth regions in human, chimpanzee and monkey faces of different expressions (HN: 93% ± 1 of overall fixations in an image-viewing trial; HR: 92% ± 1; HP: 90% ± 1; CN: 95% ± 1; CR: 94% ± 1; MN: 95% ± 1; MR: 91% ± 1). They, however, allocated lower proportion of fixations at these three key regions in dog faces (DN: 67% ± 2; DR: 67% ± 2; DP: 63% ± 1), and looked frequently at dog ears and facial hair instead.

**Fig. 4:**
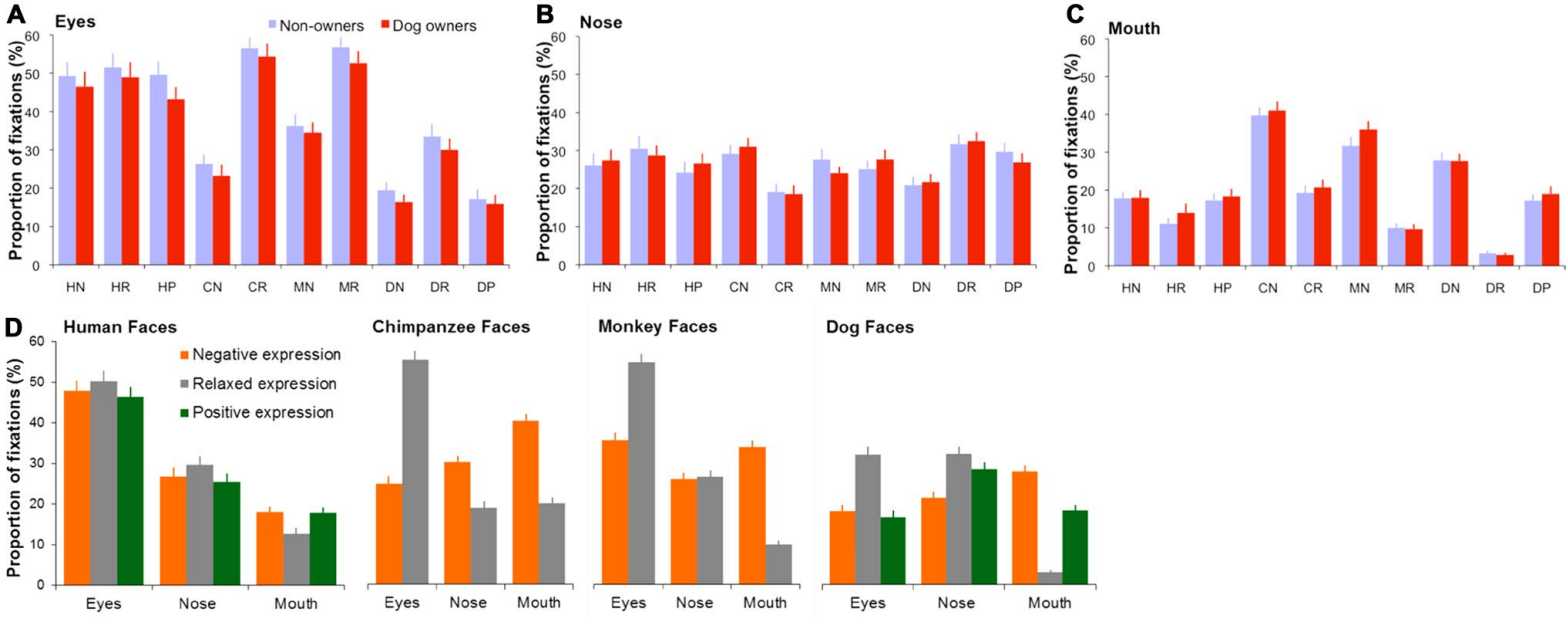
**a**–**c** Mean proportion of fixations directed at the eyes, nose and month of dog owners and non-owners when labelling human, chimpanzee, monkey and dog facial expressions. *HN* human negative, *HR* human relaxed, *HP* human positive, *CN* chimpanzee negative, *CR* chimpanzee relaxed, *MN* monkey negative, *MR* monkey relaxed, *DN* dog negative, *DR* dog relaxed, *DP* dog positive. **d** The same face-viewing fixation allocation data from all human viewers were re-grouped according to the viewed face species and facial expressions. Error bars represent SEM

A GLM analysis with proportion of fixations directed at each face region as the dependent variable was then conducted to quantitatively compare fixation distribution across different face image categories. Although dog ownership had no direct impact on gaze distribution in judging human, chimpanzee, monkey and dog facial expressions [*F*(1,1680) = .74, *p* = 0.39, *η*_p_^2^ = 0.01; Fig. 4a–c], the viewed face species and facial expressions significantly modulated the amount of fixations directed at a given local face region [face species: *F*(3,1680) = 48.59, *p* < 0.001, *η*_p_^2^ = 0.08; expression type: *F*(2,1680) = 1.04, *p* = 0.35, *η*_p_^2^ = 0.01; face regions: *F*(2,1680) = 215.67, *p* < 0.001, *η*_p_^2^ = 0.21; face species × expression type × face region: *F*(8,1680) = 18.22, *p* < 0.001, *η*_p_^2^= 0.08]. Specifically, when viewing human faces, human viewers tended to direct the highest proportion of fixations at the eyes (46–50%), followed by the nose (25–30%) and then by the mouth (13–18%) irrespective of the viewed expressions (all *p*s < 0.05; Fig. 4d). For other face species, similar pattern of gaze distribution was only observed when viewing relaxed chimpanzee and monkey faces (fixations at eyes–nose–mouth: 55%–19%–20% in chimpanzee faces; 55%–26%–10% in monkey faces); the eyes and nose in relaxed dog faces attracted indistinguishable proportion of fixations (32% vs 32%, *p *= 0.93) that was higher than those attracted by dog mouth (3%; all *p*s < 0.05).

The expressive non-human faces, on the other hand, significantly reduced fixations at the eyes (from 55 to 25%), but increased fixations at the mouth (from 10 to 40%) and nose regions (from 19 to 30%), which subsequently led to the eyes being less frequently inspected than the mouth or nose (all *p*s < 0.05). It seems that human viewers employed qualitatively similar gaze pattern to explore relaxed faces of different species, but distinctively different gaze pattern to explore expressive human and non-human faces.

Regarding the quantitative gaze allocation at each face region, for the eyes in relaxed faces, the highest proportion of fixations was directed at monkey and chimpanzee faces (~ 55%), followed by human faces (~ 50%) and then by dog faces (~ 32%; all *p*s < 0.05). For the eyes in expressive faces, the attracted proportion of fixations was monotonically decreased when the viewed face species was changed from humans to monkeys, chimpanzees and then to dogs (~ 48% → 35% → 25% → 17%; all *p*s < 0.05). In comparison with the nose in faces of different species displaying the same expression, chimpanzee nose in relaxed faces (~ 19%) and dog nose in negative faces (~ 22%) attracted fewer fixations (all *p*s < 0.05). For the mouth region in relaxed faces, chimpanzee mouth (~ 20%) and dog mouth (~ 3%) attracted the most and the least proportion of fixations respectively (all *p*s < 0.01), whereas monkey and human mouths drew similar amount of fixations (10% vs 13%; *p* = 0.09). For the mouth in expressive faces, its gaze allocation was monotonically decreased when the viewed face species was changed from chimpanzees to monkeys, dogs and then to humans (~ 40% → 34% → 28% → 18%; all *p*s < 0.05, except for indistinguishable proportion of fixations at human month and dog mouth in happy faces).

## Experiment 2: monkeys viewing heterospecific facial expressions

### Materials and methods

Monkey experiment was conducted at the Beijing Normal University. Four male adult rhesus macaques (*M. mulatta*, 5–9 kg, 5–9 years old) participated in the study. This sample size was determined based on previous research in the same field and was comparable to the published reports (e.g. Guo et al. 2003, 2006; Guo 2007; McFarland et al. 2013; Méary et al. 2014). All the monkeys were born in captivity and socially housed indoors. They grew up in large social groups, were mid-ranked individuals in the hierarchy of the colony, and were exposed to human caretakers and to their conspecifics on daily basis. The detailed experimental setup has been described in McFarland et al. (2013). Briefly, before the recording, a head restraint was implanted under aseptic conditions (for the purpose of a separate neurophysiological study) and monkeys were trained to fixate a small FP on a computer screen for a couple of seconds in exchange for juice reward. All experimental procedures were in compliance with the US National Institutes of Health Guide for the Care and Use of Laboratory Animals, and approved by the Institutional Animal Care and Use Committee of Beijing Normal University.

The presented face images were identical to those described in Experiment 1. During the recording, monkeys were seated in a primate chair with their head restrained, and viewed the display binocularly. Their horizontal and vertical eye positions were measured by EyeLink 1000 (SR Research Ltd) with 500 Hz sampling frequency, 0.25–0.5° accuracy and 0.01° root-mean-square resolution. During the calibration of eye movement signals, a small FP (0.2° diameter, 15 cd/m^2^ luminance) was displayed randomly at one of the five positions across the monitor [centre (0, 0), top (0, 7.25°), bottom (0, − 7.25°), left (− 10°, 0) and right (10°, 0)]. The monkey was required to follow the FP and maintain fixation for 1 s. After the calibration, a trial was started with an FP displayed 10° left or right to the screen centre. If the monkey maintained fixation for 1 s, the FP disappeared and an image was presented for 5 s. Unlike human viewers showing concentrated gaze allocation at the presented image, monkey viewers frequently gazed at regions outside of the screen during the image presentation. To ensure a sufficient image-viewing time for monkey viewers which was comparable to human viewers, we adopted longer image presentation duration for monkeys (5 s for monkeys vs 3 s for humans). During the free-viewing image presentation, monkeys passively viewed the images with an average image-viewing time of 3.5 s per trial. The inter-trial interval was 1 s within which monkeys received a juice reward without any specific task requirement related to the stimuli. Each monkey was tested for two identical sessions (60 images per session) separated by at least 48 h. The same eye movement analysis protocol was used for both monkey and human participants.

### Results and discussion

**Fig. 5:**
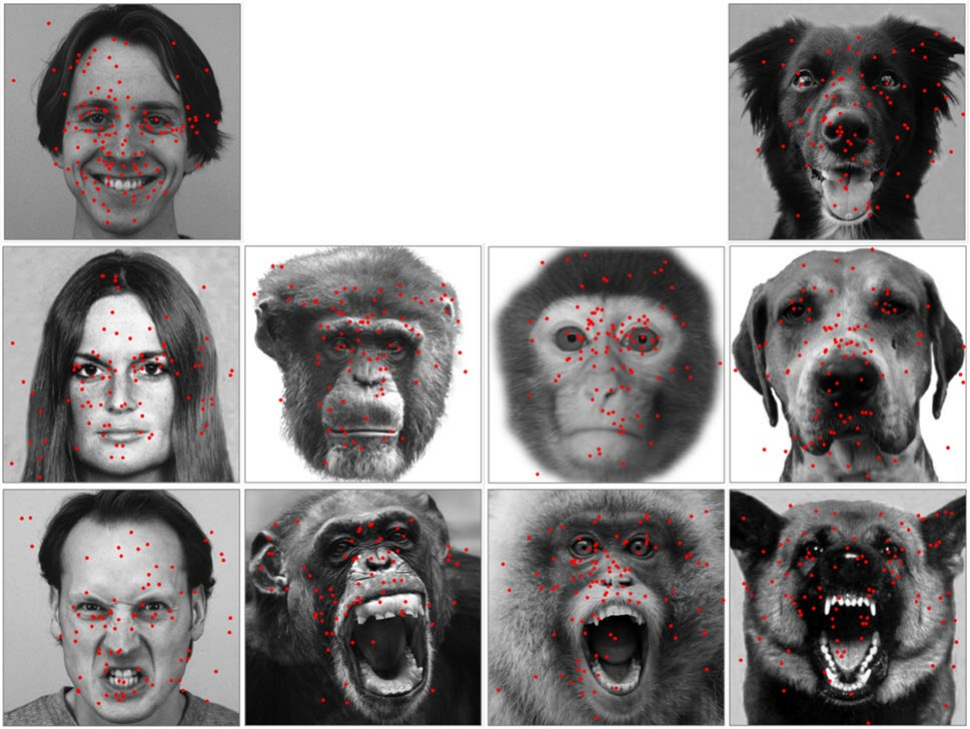
Example of overall face-viewing fixation distribution on these faces. The red dots within each face image indicate the position of each fixation sampled during this face-viewing trial across four monkey participants

Although monkey viewers frequently gazed at regions outside of the screen during the image presentation, they allocated indistinguishable number of fixations at each face image regardless of the viewed face species and facial expressions (HN: 8.10 ± 0.44; HR: 8.61 ± 0.55; HP: 8.53 ± 0.41; CN: 7.99 ± 0.58; CR: 8.93 ± 0.50; MN: 8.53 ± 0.81; MR: 9.13 ± 0.67; DN: 9.18 ± 0.42; DR: 8.93 ± 0.70; DP: 8.29 ± 0.48) [face species: *F*(3,80) = 0.42, *p* = 0.74, *η*_p_^2^ = 0.02; expression type: *F*(2,80) = 0.88, *p* = 0.42, *η*_p_^2^ = 0.03; face species × expression type: *F*(4,80) = 0.53, *p* = .72, *η*_p_^2^= 0.03]. In agreement with previous observation (Méary et al. 2014), monkeys tended to show more scattered face-viewing gaze distribution than humans (see examples in Fig. 5), and directed lower proportion of fixations at the eyes, nose and mouth in human, chimpanzee and monkey faces of different expressions (HN: 38% ± 2 of overall fixations in an image-viewing trial; HR: 88% ± 5; HP: 32% ± 2; CN: 47% ± 3; CR: 73% ± 3; MN: 62% ± 2; MR: 76% ± 3). In comparison with primate faces, they also allocated relatively fewer fixations at these three key face regions in dog faces (DN: 27% ± 1; DR: 44% ± 2; DP: 36% ± 3), and looked more frequently at dog ears and facial hair instead.

**Fig. 6:**
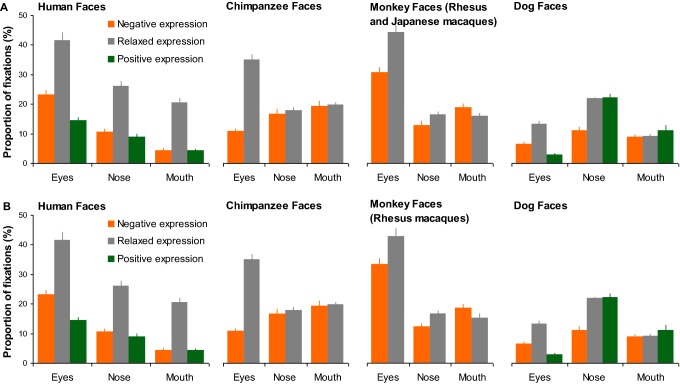
**a** Mean proportion of fixations directed at the eyes, nose and month when monkeys viewed human, chimpanzee, monkey (including both rhesus macaques and Japanese macaques) and dog facial expressions. **b** Mean proportion of fixations directed at the eyes, nose, and month when monkeys viewed human, chimpanzee, monkey (including only rhesus macaques) and dog facial expressions. Error bars represent SEM

Similar to human viewers, the viewed face species and facial expressions significantly modulated monkey viewers’ gaze allocation at internal face regions [face species: *F*(3,240) = 43.02, *p* < 0.001, *η*_p_^2^ = 0.38; expression type: *F*(2,240) = 131.61, *p* < 0.001, *η*_p_^2^ = 0.56; face region: *F*(2,240) = 66.72, *p* < 0.001, *η*_p_^*2*^ = 0.39; face species × expression type × face region: *F*(8,240) = 7.14, *p* < 0.001, *η*_p_^2^= 0.21; Fig. 6a]. Specifically, monkeys demonstrated human-like gaze distribution when viewing human faces, and directed the highest proportion of fixations at the eyes, followed by the nose and then by the mouth (41% → 26% → 20%; all *p*s < 0.05). Such pattern of gaze distribution was consistent across human faces of different expressions, although fixations at individual face regions were proportionally less in expressive faces than in relaxed faces (all *p*s < 0.05). When viewing chimpanzee and monkey faces, monkeys also directed the highest amount of fixations at the eyes (30–44%, except for negative chimpanzee faces), but slightly more fixations at mouth (16–20%) than nose region (14–18%; all *p*s < 0.05). The dog eyes (3–13%), on the other hand, attracted significantly fewer fixations than the dog nose (11–22%) and mouth (9–11%), or the eyes in human, chimpanzee and monkey faces (all *p*s < 0.05). Furthermore, unlike human viewers, monkey viewers allocated indistinguishable amount of fixations at chimpanzee mouth (17–18%) or dog mouth (9–11%) irrespective of the displayed facial expressions, although the size and shape of the mouth changed significantly between expressions in chimpanzee and dog faces.

It should be noted that in this study the monkey viewers were rhesus macaques, but the presented monkey faces were from both rhesus and Japanese macaques. To check whether these mixed monkey face stimuli would affect our findings shown in Fig. 6a, we re-analysed monkeys’ face-viewing gaze distribution by only using data from viewing conspecific rhesus macaque faces (Fig. 6b). The analysis revealed identical pattern of species- and expression-modulated gaze allocation changes as those reported above [face species: *F*(3,240) = 41.72, *p* < 0.001, *η*_p_^2^ = 0.37; expression type: *F*(2,240) = 120.71, *p* < 0.001, *η*_p_^2^ = 0.54; face region: *F*(2,240) = 69.54, *p* < 0.001, *η*_p_^2^ = 0.4; face species × expression type × face region: *F*(8,240) = 6.79, *p* < 0.001, *η*_p_^2^= 0.21], indicating that rhesus macaques showed the same gaze behaviour in viewing of rhesus macaque and Japanese macaque facial expressions.

**Fig. 7:**
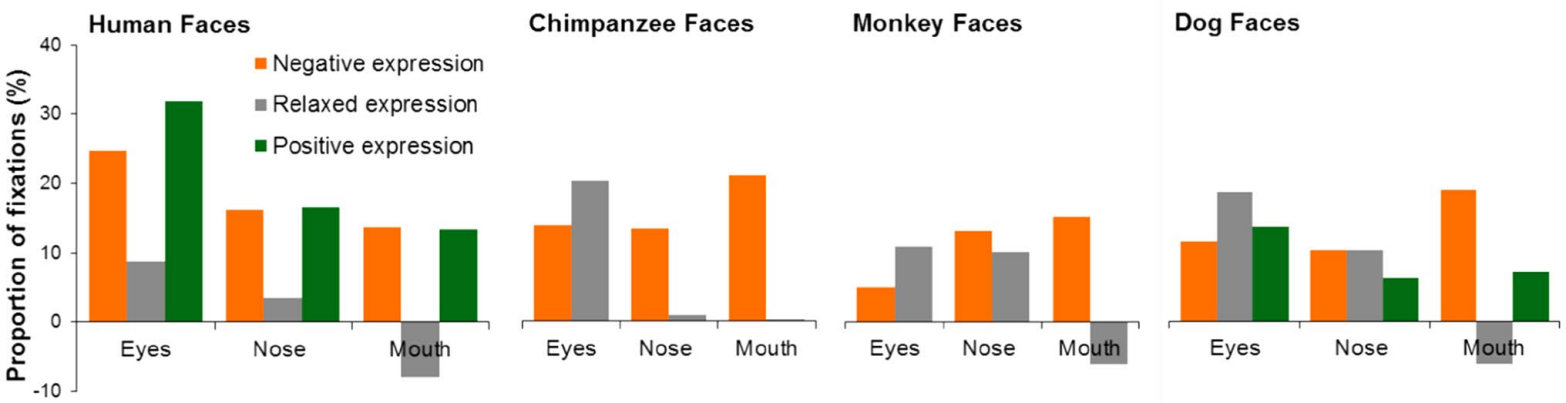
Mean differences in proportion of fixations directed at local face regions between human and monkey participants in viewing of human, chimpanzee, monkey and dog facial expressions

By visual inspection of Figs. 4 and 6, it seems that human and monkey viewers demonstrated different gaze behaviour to view the same facial expression. Even though the sample sizes of two participant species (56 humans vs 4 monkeys) were different, the direct quantitative comparison of participant species × face species × expression type × face region GLM analysis with the normalized proportion of fixations at a given face region as the dependent variable revealed clear differences in face-viewing gaze distribution between human and monkey viewers [*F*(1,1920) = 82.23, *p* < 0.001, *η*_p_^2^ = 0.04]. To present these findings informatively, for each face species and facial expression we subtracted the averaged and normalized monkey gaze allocation at a given face region from the averaged and normalized human gaze allocation at the same face region (Fig. 7). A difference larger than 0 indicates when viewing this face image, humans on average directed higher proportion of fixations at the analysed face region than monkeys, whereas a difference smaller than 0 indicates humans looked less often at this face region.

As shown in Fig. 7, in comparison with monkeys, humans tended to direct more fixations at internal facial features regardless of the viewed facial expressions and face species. Specifically, humans looked more often at the eyes (9–32% more) in all faces (all *p*s < 0.05) except for the eyes in aggressive monkey faces (*p* = 0.46), at the nose (6–16% more) in the majority of faces (all *p*s < 0.05) except for the nose in relaxed human (*p* = 0.24) and chimpanzee faces (*p* = 0.65), and at the mouth (7–21% more) in all expressive faces regardless of face species (all *p*s < 0.05). Monkeys, on the other hand, only looked more often (6–8% more) at the mouth in relaxed human, monkey and dog faces (all *p*s < 0.05). Interestingly, these differences, especially in gaze allocation at the mouth region, were affected more by the viewed facial expressions than by the viewed face species. In comparison with monkeys, humans directed similar or less amount of fixations at the mouth in relaxed faces, but higher proportion of fixations at the mouth in expressive faces irrespective of the viewed face species.

## Changes of local image saliency in faces of different species and expressions

Clearly, the viewed face species and facial expressions significantly modulated face-viewing gaze distribution for both human and monkey viewers. For instance, in comparison with human faces, expressive (especially angry/aggressive) non-human faces tended to attract fewer fixations at the eyes, but more fixations at the mouth from human viewers. As the same facial feature in faces of different species and/or different expressions varies in size (e.g. chimpanzees usually have larger mouth than humans) and low-level image salience, such as feature structure, shape, luminance intensity and contrast in local image region (e.g. exposed teeth in aggressive chimpanzee faces will enhance local contrast in mouth region), it is plausible that these changes of facial feature size and local image saliency could (at least partly) account for the observed differences in gaze allocation at a given facial feature. We examined this possibility by computing and comparing local facial feature size and local image saliency in the eyes, nose and mouth across face image categories.

For each of the face images used in this study, we first measured the proportion of the area of each facial feature (i.e. eyes, nose, and mouth) relative to the whole image. Its local image saliency was then computed in Matlab using the most widely used computational salience model of Itti and Koch (2000), with the authors’ original parameters and implementation (obtained from http://ilab.usc.edu). The model compares local image intensity, colour and orientation, combines them into a single salience map with a winner-take-all network and inhibition-of-return, and then produces a sequence of predicted fixations that scan the whole face in order of decreasing salience. We calculated the top nine salient regions within each face image because our human and monkey viewers on average made 8.5 fixations per image in face viewing. The number of predicted fixations at the eyes, nose and mouth was then normalized to the total number of predicted fixations in that face.

**Fig. 8:**
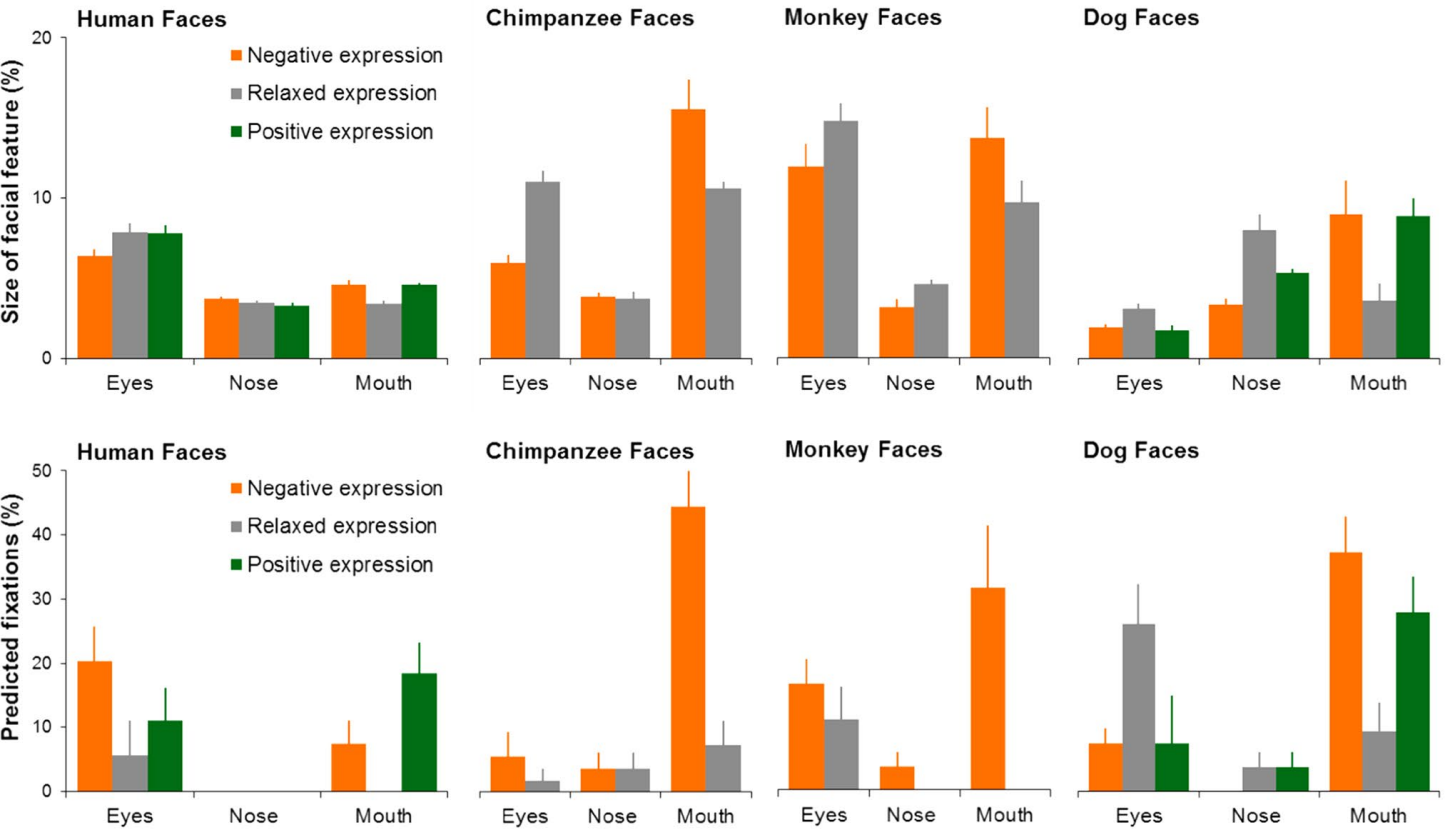
(Top row) The proportional size of the eyes, nose and mouth (relative to the whole image) in human, chimpanzee, monkey and dog faces displaying different expressions. (Bottom row) The proportion of predicted fixations at the eyes, nose, and month in human, chimpanzee, monkey and dog faces displaying different expressions. Error bars represent SEM

As shown in Fig. 8, the size of local facial features varied according to the face species and facial expressions [face species: *F*(3,180) = 48.54, *p* < 0.001, *η*_p_^2^ = 0.50; expression type: *F*(2,180) = 0.42, *p* = 0.66, *η*_p_^2^ = 0.01; face region: *F*(2,180) = 71.05, *p* < 0.001, *η*_p_^2^ = 0.49; face species × expression type × face region: *F*(8,180) = 3.26, *p* = 0.002, *η*_p_^2^= 0.15]. Specifically, the average facial feature size in chimpanzee and monkey faces was slightly larger than in human and dog faces (all *p*s < 0.001). Animals also tended to have larger mouth than humans (all *p*s < 0.001), and displaying expression could further enlarge the mouth in animal faces which was proportionally significantly larger than the mouth in expressive human faces (all *p*s < 0.001).

The computed local image saliency of different face regions also varied significantly according to the face species and facial expressions [face species: *F*(3,180) = 3.88, *p* = 0.01, *η*_p_^2^ = 0.07; expression type: *F*(2,180) = 14.59, *p* < 0.001, *η*_p_^2^ = 0.16; face region: *F*(2,180) = 40.34, *p* < 0.001, *η*_p_^2^ = 0.35; face species × expression type × face region: *F*(8,180) = 6.12, *p* = 0.002, *η*_p_^2^= 0.15]. For instance, the local image saliency around the mouth in expressive non-human faces was significantly higher than those in human faces (all *p*s < 0.001). Furthermore, facial feature size and its local image saliency tended to be correlated, in which larger facial features were normally associated with higher number of predicted fixations (two-tailed Pearson correlation, *r* = 0.47, *p* < 0.001).

The comparison between the predicted (Fig. 8) and actual fixation distribution (Figs. 4 and 6) revealed significant differences in both human [*F*(1,1860) = 271.16, *p* < 0.001, *η*_p_^2^ = 0.13] and monkey viewers [*F*(1,420) = 70.35, *p* < 0.001, *η*_p_^2^ = 0.16], suggesting the face-viewing gaze allocation in human and monkey viewers was not entirely driven by facial feature size and local image saliency. For instance, irrespective of the viewed face species and facial expressions, the relatively small eye region attracted substantially more actual fixations, especially from human viewers, than predicted (all *p*s < 0.001). However, the quantitative changes of the proportion of actual fixations at the mouth in expressive (especially negative) faces across different non-human face species showed similar trend as the changes in the mouth size and those predicted by the computational visual saliency model (Pearson correlation, all *p*s < 0.05). In comparison with the amount of fixations at the human mouth, it seems that the increased fixations at the expressive non-human animal mouth could be largely accounted for by the changes of mouth size and local image salience (e.g. changes in mouth shape, exposed teeth) between humans and non-human animals.

## General discussion

In this comparative investigation about whether the same cognitive process is used to perceive basic heterospecific facial expressions in human and monkey viewers, we presented human, chimpanzee, monkey and dog faces displaying positive (happy/playful), relaxed (neutral) and negative (angry/threatening) expressions. Human viewers showed species- and experience-dependent expression categorization accuracy. Regarding face-viewing gaze behaviour, humans and monkeys often demonstrated different gaze distribution when exploring the same face images, and the viewed face species and facial expressions could further modify their gaze allocation at the eyes, nose and mouth regions. Interestingly, such modulation of gaze behaviour was further affected by their prior experience of the viewed species. It seems that facial expression processing is species dependent, and social learning may play a significant role in discriminating even rudimentary types of heterospecific expressions.

When free labelling the perceived expressions, human viewers showed differential categorization performance for the most common types of facial expressions displayed by different species. They had the highest categorization accuracy for human faces, followed by dog and chimpanzee faces, and then by monkey faces (Fig. 2). It seems although monkeys are phylogenetically closer to humans than dogs, humans are less accurate to discriminate expressive monkey faces with which they have encountered less often in comparison with dog faces. This experience-modulated facial expression categorization extends previously reported own-race and own-species advantages in face identity recognition (Walker and Tanaka 2003; Scott and Fava 2013) and own-race advantage in facial expression recognition (Elfenbein and Ambady 2002, 2003), in which human recognition performance is biased (with increased accuracy and shortened response time) towards their own as opposed to another race’s or species’ faces. Developmental and computational studies have indicated that experience or learning history is the crucial factor for building the face feature space representation required for our successful discrimination of faces most exposed to (Pascalis et al. 2002; Dahl et al. 2014). This role of experience in judging heterospecific facial expression is further confirmed by the moderately increased recognition of dog expressions in dog owners than in non-owners, which is also in agreement with recent findings that the prolonged experience with dogs can improve the interpretation of facial and bodily emotional behaviour in dogs (Wan et al. 2012; Kujala et al. 2017).

Interestingly, humans’ expression categorization bias was also modulated by experience. When confused with relaxed faces of different species, they tended to mislabel familiar human and dog relaxed expressions as sad, but less familiar chimpanzee and monkey relaxed expressions as sad and happy.

Regarding gaze pattern associated with facial expression categorization, the viewed face species (human vs non-human faces) and expressions (relaxed vs expressive faces) had evident modulatory effect on humans’ face-viewing gaze allocation. In agreement with previous report that human spontaneous gaze behaviour in exploring relaxed conspecific and heterospecific faces is constrained by general facial configurations (Guo et al. 2010), human viewers in this study demonstrated qualitatively similar pattern of gaze distribution when viewing relaxed faces of different species, in which the eyes attracted the highest proportion of fixations, followed by the nose and then by the mouth. They, however, used distinctively different gaze patterns to explore expressive human and non-human faces. Similar to those in relaxed faces, the eye region in expressive human faces was still the most frequently attended local facial feature, suggesting its crucial role in assessing human facial expressions of emotion (Guo 2012). Unlike those in relaxed faces, the eyes in expressive non-human faces received substantial reduction in fixations; the mouth region was instead the most fixated feature for expression judgement, most likely due to both its visual saliency (e.g. significant changes in size and shape; Fig. 8) and high mobility in expressing emotions. Indeed, the facial morphology research has indicated that non-human primates, such as chimpanzees, have finer motor control of their lips than humans, and can produce more variable expressions in their mouth region (Vick et al. 2007). Consequently, the mouths in chimpanzees, monkeys and dogs are likely to be more effective in transmitting emotional cues and hence attract more fixations from human viewers. Although enlarging mouth size and increasing its image saliency (e.g. due to exposed teeth) are inherent part of emotion expression in non-human animals, future research could further untangle the relative contribution of these low-level image properties and emotional cues contained in the mouth to the increased fixations at the mouth in expressive non-human faces.

Interestingly, the changes in gaze allocation at local facial features within a given species were not strictly according to phylogenetic relatedness or prior experience alone. While eye viewing was monotonically decreased from human to monkey, chimpanzee and then to dog faces (a change related more to phylogenetic relatedness), mouth viewing was monotonically increased from human to dog, monkey and then to chimpanzee faces (a change related more to experience or exposure). It seems that both phylogenetic distance and prior experience play a role in humans’ viewing of heterospecific facial expressions.

Like human viewers, monkeys’ face-viewing gaze distribution was also significantly modulated by the viewed face species. They showed human-like gaze allocation when exploring human faces (e.g. directing the highest proportion of fixations at the eyes, followed by the nose and then by the mouth irrespective of facial expressions). Similar prolonged eye viewing was also observed on monkey and chimpanzee faces, but not on unfamiliar dog faces, suggesting that the preference of monkey’s visual system is tuned towards primate eyes. Such preference is unlikely due to low-level appearance or physical saliency in the eye region, but is more likely driven by high-level expectation of the spatial location of the eyes in facial configuration (Guo 2007) which is familiar to monkey viewers due to their prior experience (e.g. human faces) or is similar between phylogenetically close species (e.g. monkey and chimpanzee faces). This finding is in agreement with previous observation that humans, chimpanzees and rhesus monkeys showed qualitatively similar face-scanning pattern when viewing conspecific and other primates’ faces with relaxed expressions (Guo et al. 2003; Kano and Tomonaga 2010; Méary et al. 2014; see also Dahl et al. 2009), indicating the homologous nature of face-viewing gaze behaviour in primates which is probably driven by the crucial role of eye detection in face reading and associated behavioural responses (e.g. towards the approach of a potential mate, competitor or predicator) (Emery 2000).

The viewed facial expressions further modulated monkeys’ gaze distribution. The eyes in expressive faces (especially in negative monkey and chimpanzee faces) tended to attract fewer fixations in comparison with the eyes in relaxed faces, implying an avoidance gaze behaviour in monkeys’ viewing of aggressive conspecific and heterospecific faces (gaze aversion generally indicates anxiety and submissiveness; Deaner et al. 2005). Interestingly, the infrequently experienced chimpanzee faces showed less modulatory effect on monkeys’ gaze behaviour than the frequently (or daily) experienced human faces (e.g. monkeys directed indistinguishable amount of fixations at chimpanzee mouth even its size and shape changed significantly between expressions). It seems that prior experience also play a role in monkeys’ viewing of heterospecific faces.

It should be noted that the presented monkey faces in this study were from both rhesus and Japanese macaques. Although we do not expect a clear difference in the facial display of neutral and angry/threatening expressions between two macaca species, future research could systematically examine the potential differences in facial muscle movements for displaying a wider range of expressions among macaques (e.g. Rhesus macaque vs Japanese macaque vs Barbary macaque), possibly via facial action coding system. We did not include positive chimpanzee and monkey expressions in our presentation; future research could also examine interspecies emotional understanding of those well-defined primate facial displays in positive social contact, such as play faces in chimpanzees. Furthermore, the face images used in this study were in full frontal view with direct gaze. It is well established that direct gaze represents a strong signal of threat for macaque monkeys (Dunbar 1991), and viewers often look at the face briefly to reduce direct gaze contact, particularly when encountering faces with angry/aggressive expression or without clear approaching signals (e.g. McFarland et al. 2013). Hence, it is plausible that monkey viewers may interpret ‘neutral/relaxed’ frontal-facing faces differently in comparison with human viewers, especially in viewing of conspecific faces. Future research could replicate this study by using faces of different species and different expressions in mid-profile or profile view.

Nevertheless, the current study represents an important step forward in our understanding of how primates read and understand heterospecific facial displays of emotion. Comparing face-viewing gaze allocation between human and monkey viewers revealed both species-general and species-specific forms of facial communication. The common characteristics is that both species showed the persistent and prolonged eye viewing for primate faces (i.e. human, chimpanzee and monkey faces) irrespective of facial expressions, which enables the constant retrieval of subtle emotional and intentional (e.g. gaze direction) information conveyed by the eye region in primates.

Furthermore, the significance of face signalling and face reading in primates’ social interaction, and very similar cortical mechanism in face processing would suggest a comparable visual sensitivity and recognition performance of conspecific and heterospecific facial expressions across primates (Leopold and Rhodes 2010). Although humans and monkeys may show certain degree of species-invariant innate bias or visual preference to basic facial expressions of emotion, the more accurate or effective cognitive process and fine interpretation of heterospecific expressions are likely subject to social or experiential learning process, as in this study we observed an experience-dependent expression categorization accuracy in humans, and a species-dependent gaze distribution in both human and monkey viewers. It is possible that (1) emotion expression is species specific, even for the rudimentary positive and negative affects (such as those used in this study); (2) across the species, the function of facial muscles of non-conspecifics may not match their equivalent function in conspecific face. Therefore, our findings would challenge the view of applying human-equivalent ‘universality’ in the process of facial expressions in interspecies emotion perception.

Indeed, it has been proposed that the lifelong social learning is crucial to shape our emotion perception and emotional responses, such as cognitive representations of emotional triggers, experiences and associated coping responses (Ekman and Cordaro 2011). However, as the basic emotional expressions and responses are evolutionarily shaped, biologically pre-wired and psychologically primitive (Darwin 1872; Schirmer and Adolphs 2017), it could be argued that there is existence of an inborn ‘open appraisal program’ (Ekman and Cordaro 2011) for processing these universal emotional expressions (such as facial expressions) irrespective of species, and social learning will quantitatively increase the efficiency and accuracy of expression recognition. In other words, perceiving conspecific and heterospecific facial expressions is influenced but not totally constructed by experience. Future research could explicitly examine this hypothesis.
